# Large-scale analysis of putative plasmids in clinical multidrug-resistant *Escherichia coli* isolates from Vietnamese patients

**DOI:** 10.3389/fmicb.2023.1094119

**Published:** 2023-05-31

**Authors:** Quang Huy Nguyen, Thi Thu Hang Le, Son Thai Nguyen, Kieu-Oanh Thi Nguyen, Dong Van Quyen, Juliette Hayer, Anne-Laure Bañuls, Tam Thi Thanh Tran

**Affiliations:** ^1^University of Science and Technology of Hanoi, Vietnam Academy of Science and Technology, Hanoi, Vietnam; ^2^LMI DRISA, IRD-USTH, Hanoi, Vietnam; ^3^Institute of Biotechnology, Vietnam Academy of Science and Technology, Hanoi, Vietnam; ^4^UMR MIVEGEC, University of Montpellier-IRD-CNRS, Montpellier, France

**Keywords:** *Escherichia coli*, putative plasmid, carbapenem resistance, horizontal gene transfer, whole-genome sequencing

## Abstract

**Introduction:**

In the past decades, extended-spectrum beta-lactamase (ESBL)-producing and carbapenem-resistant (CR) *Escherichia coli* isolates have been detected in Vietnamese hospitals. The transfer of antimicrobial resistance (AMR) genes carried on plasmids is mainly responsible for the emergence of multidrug-resistant *E. coli* strains and the spread of AMR genes through horizontal gene transfer. Therefore, it is important to thoroughly study the characteristics of AMR gene-harboring plasmids in clinical multidrug-resistant bacterial isolates.

**Methods:**

The profiles of plasmid assemblies were determined by analyzing previously published whole-genome sequencing data of 751 multidrug-resistant *E. coli* isolates from Vietnamese hospitals in order to identify the risk of AMR gene horizontal transfer and dissemination.

**Results:**

The number of putative plasmids in isolates was independent of the sequencing coverage. These putative plasmids originated from various bacterial species, but mostly from the *Escherichia* genus, particularly *E. coli* species. Many different AMR genes were detected in plasmid contigs of the studied isolates, and their number was higher in CR isolates than in ESBL-producing isolates. Similarly, the *bla*_KPC-2_, *bla*_NDM-5_, *bla*_OXA-1_, *bla*_OXA-48_, and *bla*_OXA-181_ β-lactamase genes, associated with resistance to carbapenems, were more frequent in CR strains. Sequence similarity network and genome annotation analyses revealed high conservation of the β-lactamase gene clusters in plasmid contigs that carried the same AMR genes.

**Discussion:**

Our study provides evidence of horizontal gene transfer in multidrug-resistant *E. coli* isolates via conjugative plasmids, thus rapidly accelerating the emergence of resistant bacteria. Besides reducing antibiotic misuse, prevention of plasmid transmission also is essential to limit antibiotic resistance.

## 1. Introduction

The rapid increase of antibiotic-resistant bacteria, which is the consequence of excessive and inappropriate use of antibiotics (Kahlmeter et al., 2003; Costelloe et al., 2010), is a tremendous public health issue because it hinders the proper treatment of infections, thus increasing morbidity and mortality and also healthcare costs (Cosgrove and Carmeli, 2003). It was estimated that in 2019, antimicrobial resistance (AMR) was associated with ~4.95 million deaths worldwide and that AMR bacteria directly caused 1.27 million deaths. *Escherichia coli* is one of the six leading AMR bacteria associated with more than 800,000 deaths (Antimicrobial Resistance Collaborators, 2022). *E. coli*, a Gram-negative bacterial species, is found in the lower intestine of humans and plays a vital role as commensal bacterium, but can also cause urinary tract infections, sepsis and meningitis (McNally et al., 2013), and is the leading cause of community and hospital infections. Many studies have highlighted the AMR threat in healthcare and agricultural settings worldwide, but particularly in low- and middle-income countries (Nhung et al., 2016; Yamasaki et al., 2017; Tran et al., 2019). Even if antibiotic resistance is a global issue, Asia remains the major source of resistance in the world. Almost 35% of the emerging infectious diseases identified in Asia between 1940 and 2004 correspond to the emergence of a new pattern of antimicrobial drug resistance (Jones et al., 2008; Horby et al., 2013). South East Asia including Vietnam is considered as a hot spot for AMR emergence because of the possible access to antimicrobials for humans without prescription despite the regulations and the high antimicrobial usage for livestock (Mao et al., 2015; Carrique-Mas et al., 2020). Despite the National Action Plan to combat AMR implemented since 2013, Vietnam is still heavily affected by the emergence and rapid spread of bacteria, resistant to many antibiotics. A recent large-scale study on 3,153 multidrug-resistant *E. coli*, *Klebsiella pneumoniae* and *Acinetobacter baumannii* isolates from two intensive care units (ICU) reported a high prevalence of AMR genes and evidence of extensive transmission between ICU patients in Vietnam (Roberts et al., 2022).

The spread of antibiotic resistance is mostly driven by horizontal gene transfer via conjugative plasmids (von Wintersdorff et al., 2016). Majority of carbapenem-resistant *E. coli* strains harbor *bla*_NDM-1_ in China (Zhang et al., 2017) and Vietnam (Tran et al., 2015), which emphasizes regionally dissemination of carbapenem-resistant *Enterobacteriaceae* depending on the horizontal transfer of their plasmid-mediated genes. Among the *Enterobacteriaceae, E. coli* can incorporate extracellular plasmids ‘naturally’ without special treatment (Tsen et al., 2002). According to the NCBI database (accessed on May 31, 2022), up to 17 different types of native plasmids can be found in the *E. coli* genome and they represent up to 14% of *E. coli* genetic material. Unlike chromosomes, plasmids can exist in multiple copies that increase the conjugation frequency and allow the rapid spread of AMR genes within microbial communities (Dimitriu et al., 2021). Plasmid-encoded genes are often carried in complex structures and exist in a variety of plasmid types (Hirabayashi et al., 2021). Given the vital role of plasmids in the acquisition and dissemination of AMR genes, it is important to thoroughly study their characteristics in multidrug-resistant clinical isolates. In the present study, we performed a large-scale analysis of putative plasmids using whole-genome sequencing data retrieved from several published studies on extended-spectrum beta-lactamase (ESBL)-producing and carbapenem-resistant (CR) *E. coli* isolates from Vietnamese patients. The presence of AMR genes on putative plasmids was investigated to assess the risk of transmission particularly of genes associated with resistance to carbapenems.

## 2. Materials and methods

### 2.1. Whole-genome sequencing data of multidrug-resistant *Escherichia coli* isolates in Vietnam

In this analysis, we searched all genomic studies investigating multidrug-resistant *E. coli* isolated from clinical samples in Vietnam. We eliminated studies that did not perform whole-genome sequencing using Illumina platforms or did not publish raw sequencing data, leaving 3 studies for inclusion. Raw sequencing data of 751 multidrug-resistant *E. coli* strains previously published by Roberts et al. (2022) (ENA Bioproject: PRJEB29424 and PRJEB28400), Hirabayashi et al. (2021) (provided by the authors), and Tuan-Anh et al. (2020) (ENA Bioproject PRJEB39354) were downloaded from the European Nucleotide Archive (ENA) database. Sixteen samples were from environmental surfaces (environmental isolates) and were collected at the National Hospital for Tropical Diseases, Hanoi, Vietnam. Among the other 735 clinical samples, 720 of the 721 isolates from the study by Roberts et al. (2022) were collected in two ICUs in Hanoi: 275 from the Bach Mai Hospital and 445 from the National Hospital for Tropical Diseases. One duplicate isolate (NHP1391) was excluded from the analysis. The two isolates (MH13 and MH17) from the study by Hirabayashi et al. (2021) were from the Military Hospital 103 in Hanoi, and the 27 isolates from the study by Tuan-Anh et al. (2020) were from the Children’s Hospital 1 in Ho Chi Minh city. Two *E. coli* isolates (TN1393 and XP817) were from our laboratory (NCBI SRA BioProject: PRJNA857185) and were from two patients hospitalized in the Saint Paul Hospital and the 108 Military Central Hospital, respectively. Most of the studied samples were collected between 2017 and 2019, except three samples (MH13, TN1393 and XP817) collected in 2012 and 2013. Each isolate is described in Supplementary Table S1.

### 2.2. Antimicrobial resistance profiling

The *E. coli* isolates from the study by Roberts et al. (2022) were classified into two phenotypes, ESBL-producing isolates and CR isolates, in function of their growth on selective media. The phenotype classification of the other isolates was based (i) on the antimicrobial susceptibility test results extracted from the studies by Hirabayashi et al. (2021) and Tuan-Anh et al. (2020), and (ii) for the two isolates from our laboratory (TN1393 and XP817), on antimicrobial susceptibility testing performed using ampicillin, amoxicillin-clavulanic acid, amikacin, piperacillin, ticarcillin, ticarcillin-clavulanic acid, cefalexin, cefoxitin, ceftazidime, colistin, fosfomycin, ofloxacin, imipenem, penicillin, and trimethoprim-sulfamethoxazole and the Kirby Bauer disk diffusion method, as previously described (EUCAST, 2020). In total, 4/751 strains were classified as non-ESBL-producing strains (see also Supplementary Table S1).

### 2.3. Genomic DNA extraction and Illumina sequencing

Genomic DNA (gDNA) of the two isolates from our laboratory was extracted with the Bacterial Genomic DNA Isolation Kit (Norgen Biotek Corp., Thorold, Ontario, Canada) following the manufacturer’s instructions. The gDNA samples were sheared randomly into small fragments with Covaris S/E210 or Bioruptor to prepare paired-end fragment libraries that were sequenced using an Illumina HiSeq 4,000 system at the Beijing Genomics Institute (Shenzhen, China). According to the information in the articles, gDNA was extracted with the QIACube and QIAamp 96 DNA QIACube HT kit (Qiagen, Hilden, Germany) and sequenced on a Illumina HiSeq X10 machine (Roberts et al., 2022); gDNA was extracted with the Wizard Kit (Promega) and sequenced on a Illumina MiSeq platform (Tuan-Anh et al., 2020); gDNA was extracted with the phenol-chloroform method and sequenced on a MiSeq (MH13 isolate) and on a HiSeq 4000 (MH17 isolate) apparatus (Hirabayashi et al., 2021).

### 2.4. Bioinformatics analysis

**Figure 1:**
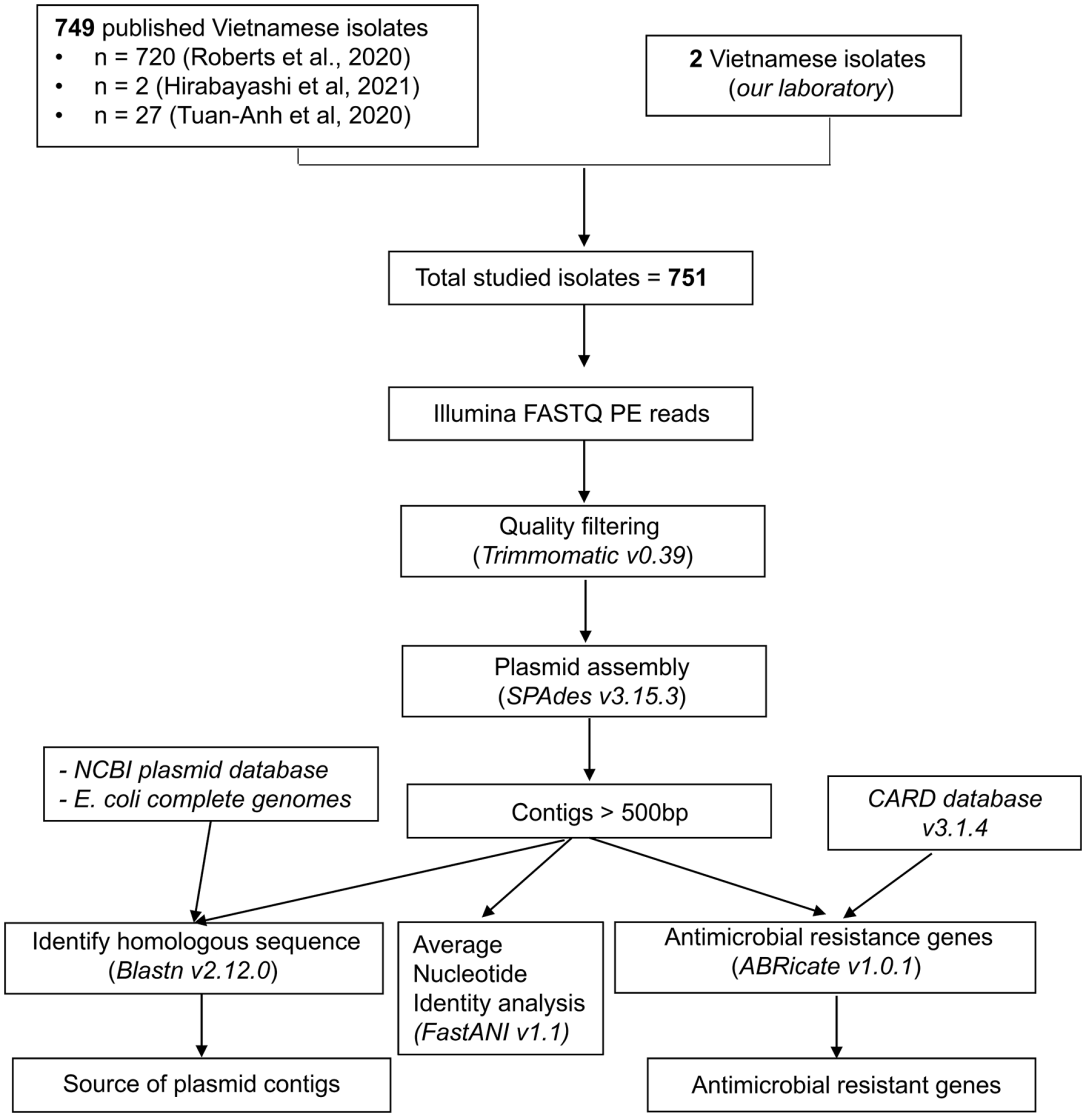
Bioinformatics workflow for plasmid analysis using raw sequencing data from multidrug-resistant *E. coli* isolates.

Raw whole-genome sequencing data were analyzed using the bioinformatics workflow described in Figure 1.

#### 2.4.1. De novo assembly of whole-genome sequencing data

The paired-end raw reads were trimmed with Trimmomatic v0.39 (Bolger et al., 2014), using a Phred threshold score < 20, and the following parameters: SLIDINGWINDOW:4:20 LEADING:20 TRAILING:20 MINLEN:50. Trimmed reads were assembled into contigs using SPAdes *de novo* assembler v3.14.1 (Bankevich et al., 2012) with default parameters for genome assembly and with the ‘plasmid’ flag (plasmidSPAdes) for assembling plasmids (Antipov et al., 2016). Contigs <500 bp were discarded.

#### 2.4.2. Identification of plasmid contigs and their origin

The contigs assembled with plasmidSPAdes were aligned to the NCBI RefSeq plasmid database (accessed on July 7, 2022) using BLASTn v2.12.0 (Altschul et al., 1990). A contig matched to the nearest neighbor (the highest BLAST bit score) in the plasmid database with a minimal identity of 95% and a minimal query coverage of 50% was defined as “putative plasmid contig.” Then, the remaining unassigned contigs were aligned against the 2,174 complete *E. coli* genomes available in the NCBI genome database (accessed on May 31, 2022) with BLASTn v2.12.0, and the previous thresholds were used to identify chromosomal contamination in the plasmidSPAdes assembly.

The taxonomic lineages of the plasmid homologs were obtained using the NCBI Taxonomy database (accessed on July 24, 2022). The plasmid homolog sequences were extracted from the plasmid databases using seqtk v1.3 (Li, 2013). The average nucleotide identity (ANI) between plasmid homologs was estimated using FastANI v1.1 (Jain et al., 2018). All pairs with ANI values <95% were excluded. The plasmid network was then built with Cytoscape v3.9.0 (Shannon et al., 2003).

#### 2.4.3. Identification of chromosome contigs

For each isolate, contigs from the SPAdes assembly were aligned to their putative plasmid contigs from the plasmidSPAdes assembly using BLASTn v2.12.0 (Altschul et al., 1990). All contigs matched to plasmid contigs with a minimum identity of 99% and a minimum query coverage of 99% were filtered out, and the remaining contigs were identified as “chromosome contigs.”

#### 2.4.4. Detection of antimicrobial resistance genes

AMR genes carried on contigs were identified using ABRicate v1.0.1 (Seemann, n.d.-a) and the CARD database v3.2.4 (Jia et al., 2017) and the following thresholds: 80% of minimum identity and 50% of minimum coverage. AMR genes were grouped into AMR gene families and drug classes using the CARD database v3.2.4. Contigs were functionally assessed with Rapid Annotations using Subsystem Technology (RAST) version 2.0 (Aziz et al., 2008).

#### 2.4.5. Construction of the sequence similarity network

Pairwise alignments between putative plasmid contigs were carried out using BLASTn in the blastall (“BLAST all against all”) program with an e-value threshold of 1 × 10^−10^ (Camacho et al., 2009). The BLAST best hit for each pairwise alignment was determined. Alignments with identity <99% and minimum coverage <90% and also repetitive alignments were filtered out. The remaining best alignments were used to build the sequence similarity network with Cytoscape v3.9.0 (Shannon et al., 2003).

#### 2.4.6. Multilocus sequence typing analysis

Multilocus sequence typing (MLST) data for the isolates were obtained from the published papers, or determined from the assembled genomes using mlst v2.22.0 (Seemann, n.d.-b) with the *E. coli* species scheme.

### 2.5. Statistical analyses

Statistical analyses and data visualization were done with R version 4.0.4 (R Core Team, 2016). The numbers of putative plasmid contigs and of AMR genes were compared in resistance phenotype groups with the Kruskal-Wallis test followed by the Dunn’s post-hoc tests for multiple pair comparisons using the kruskal.test function and the dunn.test function. The Fisher’s exact test was used to compare AMR genes between CR isolates and ESBL-producing isolates through fisher.test function. All *p*-values from multiple testing were corrected with the Benjamini-Hochberg method, and adjusted *p-*values <0.05 were considered significant using the p.adjust function. Correlation plots, box plots and bar plots were generated using the ggplot function from the ggplot2 package version 3.4.0. Binary heatmaps were constructed with the Heatmap function from the ComplexHeatmap package version 2.9.4 (Gu et al., 2016).

## 3. Results

### 3.1. Characteristics of putative plasmid contigs

**Figure 2:**
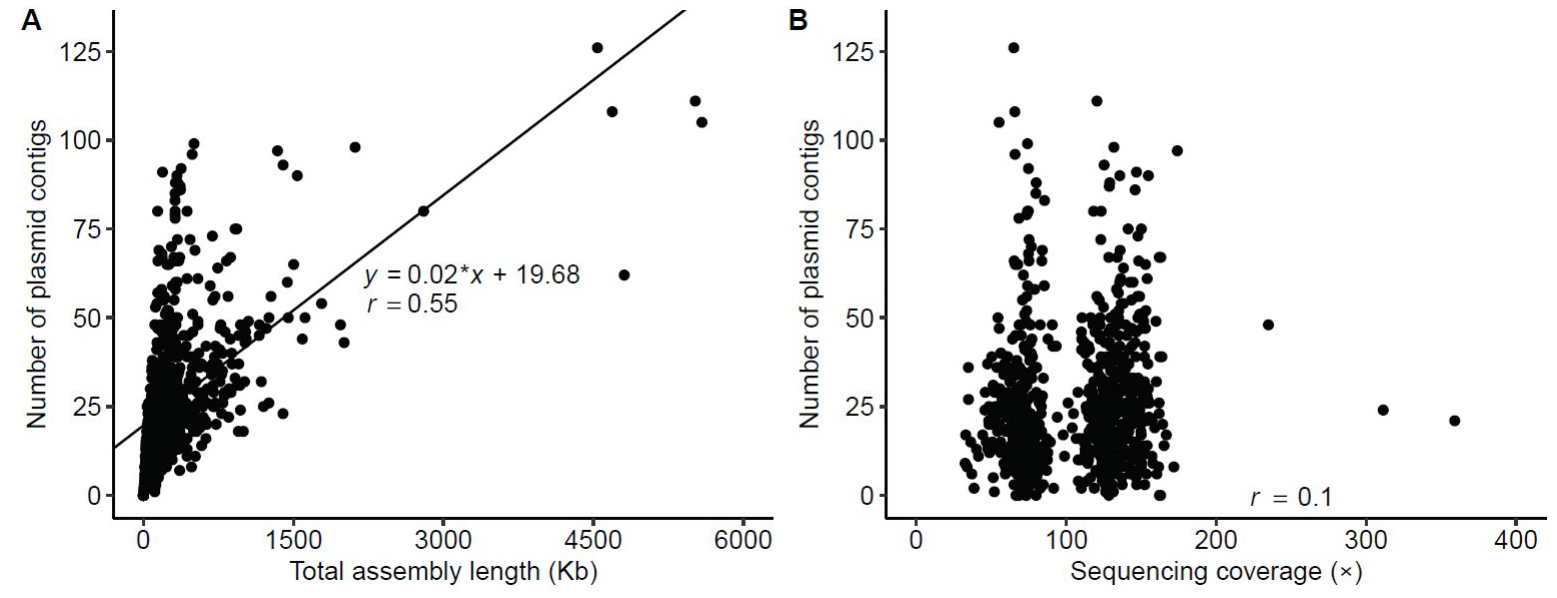
Statistics of contigs assembled using the plasmidSPAdes assembler. **(A)** Correlation of the number of plasmid contigs ≥500 bp with the total assembly length. **(B)** Relationship between number of contigs with length ≥ 500 bp and sequencing coverage. Correlations were assessed using a linear regression model; *r*, Pearson’s correlation coefficient.

The number of assembled plasmid contigs, using plasmidSPAdes, hugely varied among the 751 isolates, from 0 to 126 (median = 22, mean ± SD = 26 ± 19). In 8/751 isolates, no plasmid contig was identified. As expected, a moderate positive correlation was found between plasmid contig number and total assembly length (Pearson’s correlation coefficient*, r* = 0.55) (Figure 2A; Supplementary Table S1). Conversely, no correlation was found between plasmid contig number and sequencing coverage (*r* = 0.1) (Figure 2B; Supplementary Table S1), suggesting that the number of plasmid contigs in an isolate was linked to the presence of plasmids rather than to the sequencing coverage. Moreover, the number of plasmid contigs was higher in CR isolates (*n* = 197) than in ESBL-producing isolates (*n* = 550) (*p* = 0.008) and non-ESBL-producing isolates (*n* = 4) (*p* = 0.041) (Supplementary Figure S1). However, the highest number of plasmid contigs was found in an ESBL-producing strain (*n* = 126).

**Figure 3:**
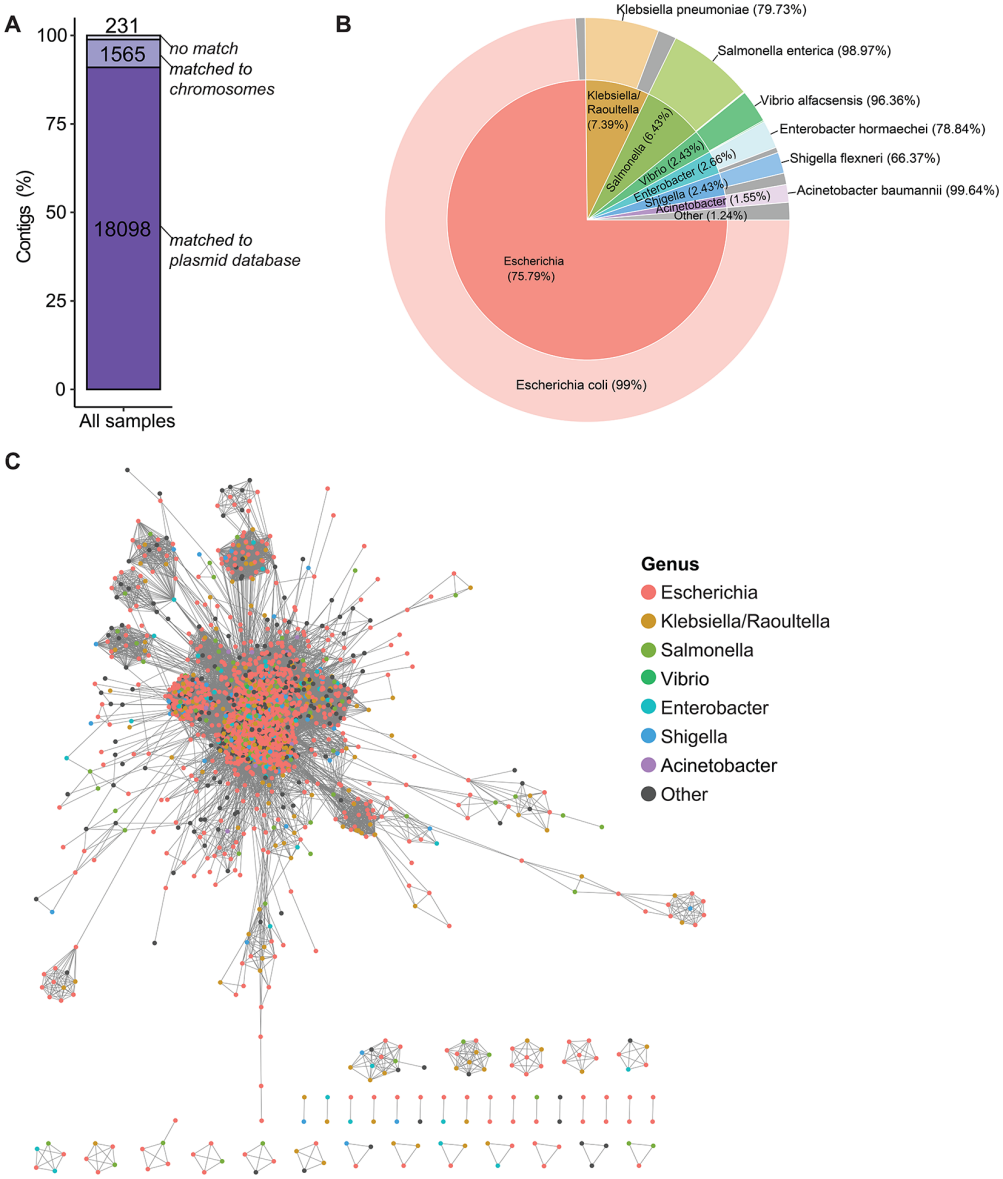
Origin of the putative plasmid contigs. **(A)** BLASTn homology search of contigs against the plasmid database and the complete *E. coli* genomes using the following thresholds: 95% of minimum identity and 90% of minimum query coverage. The numbers in the bars indicate the number of contigs. **(B)** Pie chart showing the percentages of plasmid homologs that matched to the putative plasmid contigs grouped by host genus and host species. “Other” (in gray): other species/genera with matching <1%. **(C)** Clustering of plasmid homologs by average nucleotide identity with a cut-off value of 95%. Each node represents a plasmid homolog and is color-coded in function of the host genus.

Among the 19,894 plasmid contigs identified with plasmidSPAdes in the studied isolates, 18,098 (90.97%) were matched in the plasmid database (putative plasmid contigs) and 1,565 (7.87%) were matched to the *E. coli* chromosome with at least 95% sequence identity (Figure 3A). The other 231 contigs (1.16%) could not be matched (i.e., unassigned contigs). However, for 223/231 unassigned contigs, at least one homolog was found in the plasmid database or in the *E. coli* genome database, but with a low BLAST identity (between 70 and 93%). For the other unassigned contigs, a BLASTn homology search against the non-redundant (nr) nucleotide database found that four contigs shared high similarity with the *A. baumannii* chromosome (sequence identity >98%), one contig shared 96% of identity with an *Escherichia* phage, and three contigs could not be aligned to any sequence.

Next, the closest match of the putative plasmid contigs in the plasmid database, which was considered as the plasmid homolog, was investigated. Plasmid homologs were mostly from the *Escherichia* genus (75.79%), followed by the *Klebsiella/Raoultella, Salmonella, Vibrio, Enterobacter, Shigella*, and *Acinetobacter* genera. These genera mostly belong to the *Enterobacteriaceae* family, with the exception of *Vibrio* that belongs to the *Vibrionaceae* family and of *Acinetobacter* that belongs to the *Moraxellaceae* family. Each genus was dominated by a major species (between 66.37 and 99.64% of plasmid homologs for that genus) (Figure 3B). This indicates that plasmids could be shared between genera, but the transmissions of plasmids are more frequently observed within the same species than those between distinct species. A set of 2,331 plasmid types that represented all plasmid homologs was used for the ANI analysis based on whole plasmid alignments to identify the genetic relatedness among plasmid homologs. This analysis revealed that 1,955/2,331 plasmids from different genera were clustered together with an ANI value >95%, indicating that these plasmid homologs were very closely related. Conversely, 120/2,331 plasmids were in smaller clusters, and 256/2,331 plasmids did not share any similarity with the other plasmids (Figure 3C).

### 3.2. Antimicrobial resistance genes in the putative plasmid contigs

**Figure 4:**
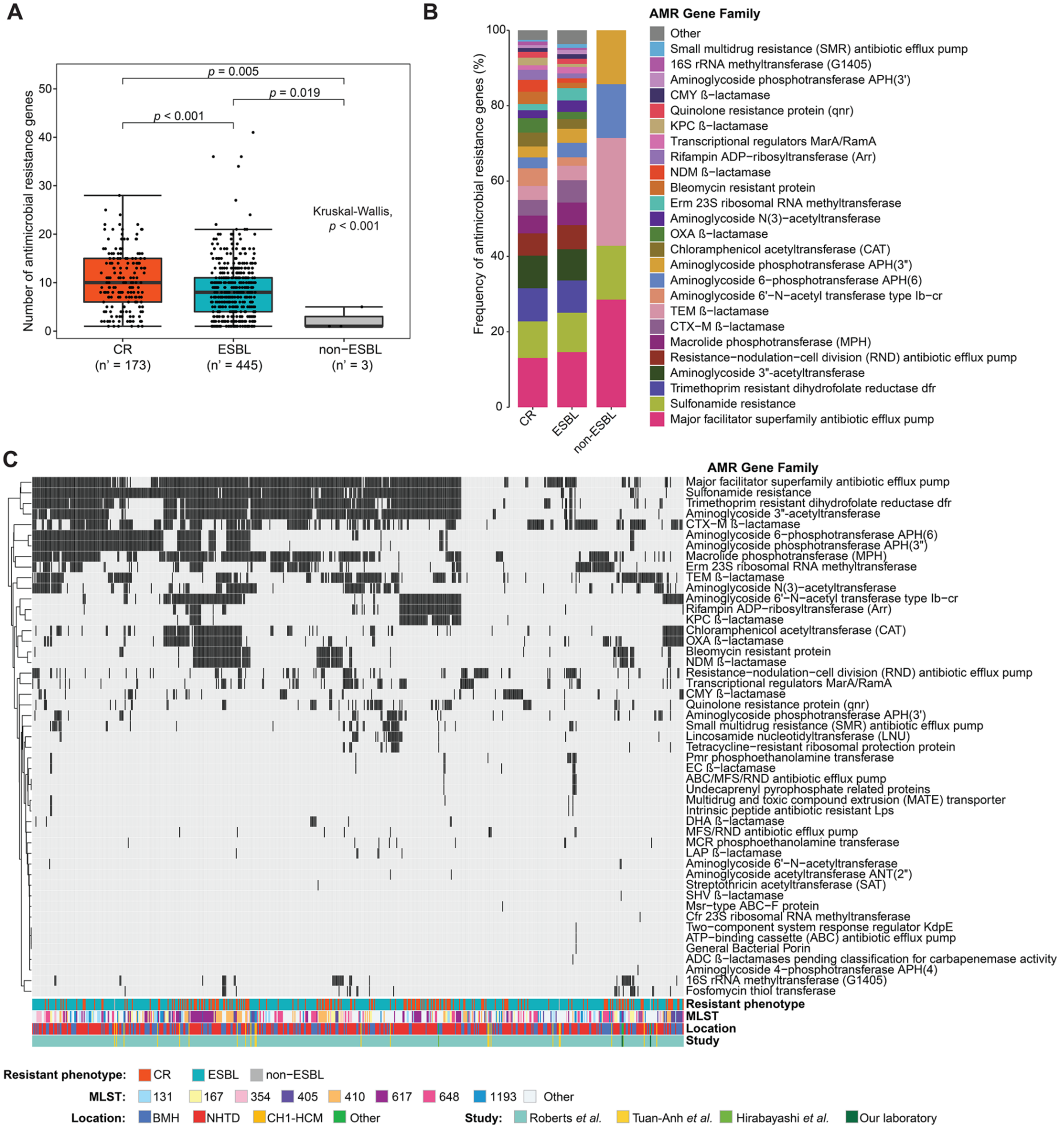
Antimicrobial resistance (AMR) genes in putative plasmid contigs. **(A)** Box plot showing the number of AMR genes detected in putative plasmid contigs of isolates grouped in function of their resistance phenotype; n’, number of isolates with the putative plasmid contigs carrying AMR genes. *P*-value calculated with the Kruskal-Wallis test followed by the Dunn’s *post-hoc* test and corrected for multiple testing with the Benjamini-Hochberg method. **(B)** Frequency of AMR genes, classified into AMR gene families, detected on putative plasmid contigs in carbapenem-resistant (CR) isolates, extended-spectrum beta-lactamase (ESBL)-producing isolates and non-ESBL-producing isolates. AMR genes with frequency < 1% were merged in the “Other” group. **(C)** Heatmap showing the presence (black) and absence (gray) of AMR gene families across isolates. Only isolates carrying at least one AMR gene were included. Hierarchical clustering was performed using Euclidean distances. The color bars at the bottom of the heatmap indicate the resistance phenotype, sequence type (MLST), location, and study from which the sequencing data of the isolates were taken. BMH, Bach Mai Hospital; NHTD, National Hospital for Tropical Diseases; CH1-HCM, Children’s Hospital 1-Ho Chi Minh city.

In total, 5,554 plasmid-carried AMR genes were found in 83.98% (624/743) of isolates with plasmids. Among these genes, 99.06% (5,502/5,554) were detected in contigs that were matched in the plasmid database (Supplementary Table S2) and the other 52 AMR genes were detected in contigs that matched to chromosomes and in unassigned contigs. More than 60% of AMR genes identified in putative plasmid contigs belonged to the major facilitator superfamily antibiotic efflux pump, sulfonamide resistance, trimethoprim resistant dihydrofolate reductase, aminoglycoside acetyltransferase ANT(3″), resistance-nodulation-cell division antibiotic efflux pump, macrolide phosphotransferase, CTX-M β-lactamase, and TEM β-lactamase families (Supplementary Table S2 and Supplementary Table S2). The mean number of AMR genes detected in plasmid contigs for each isolate was nine. Overall, the number of AMR genes in plasmid contigs was higher in CR isolates than ESBL-producing isolates (*p* < 0.001). As expected, non-ESBL-producing isolates, which had a lower resistance phenotype, had fewer AMR genes than CR and ESBL-producing isolates (Figure 4A). Both CR and ESBL-producing isolates harbored many different plasmid-carried AMR genes belonging to >40 different AMR gene families. Conversely, only five AMR gene families [i.e., major facilitator superfamily antibiotic efflux pump, sulfonamide resistance, TEM β-lactamase, aminoglycoside 6-phosphotransferase APH (6), aminoglycoside 3″-phosphotransferase were found in non-ESBL-producing isolates]. Although CR and ESBL-producing isolates showed similar frequencies of AMR gene families across the putative plasmid contigs, the frequency of genes encoding OXA, NDM and KPC β-lactamases, which are implicated in carbapenem resistance, varied between CR and ESBL-producing isolates (Figure 4B and Table 1). The presence/absence of AMR genes classed into AMR gene families was further investigated in each isolate. AMR genes belonging to the major facilitator superfamily, sulfonamide resistance, trimethoprim resistant dihydrofolate reductase, and aminoglycoside acetyltransferase ANT(3″) families were highly represented in the studied isolates. While there was no clear separation between groups on the binary heatmap of AMR gene family across the studied strains, the presence of AMR genes in isolates was not likely associated with their resistance phenotype, sequence type (MLST), location, and study. However, isolates belonging to the sequence type 617 (ST617) could be grouped in clusters on the basis of their AMR gene profile, and these clusters included mainly CR isolates (Figure 4C).

**Table 1:** Presence of AMR genes associated with resistance to carbapenems on putative plasmid contigs.

AMR gene	CRE isolates (*n* = 197)		ESBL isolates (*n* = 550)		Fisher’s exact test	
	No. isolates carry AMR genes	Occurrence (%)	No. isolates carry AMR genes	Occurrence (%)	Normal *P*	Adjusted P*
CTX-M-27	19	9.64	87	15.82	0.033	0.103
KPC-2	36	18.27	31	5.64	<0.0001	<0.0001
NDM-5	46	23.35	37	6.73	<0.0001	<0.0001
NDM-1	4	2.03	1	0.18	0.019	0.069
NDM-4	3	1.52	4	0.73	0.388	0.840
NDM-7	3	1.52	2	0.36	0.118	0.324
OXA-1	44	22.34	52	9.45	<0.0001	<0.0001
OXA-9	1	0.51	1	0.18	0.458	0.840
OXA-23	0	0.00	1	0.18	1	1
OXA-339	0	0.00	1	0.18	1	1
OXA-70	1	0.51	0	0.00	0.264	0.645
OXA-48	12	6.09	7	1.27	0.001	0.004
OXA-181	10	5.08	7	1.27	0.004	0.019
OXA-928	0	0.00	1	0.18	1	1
SHV-12	0	0.00	1	0.18	1	1
TolC	1	0.51	4	0.73	1	1
marA	13	6.60	44	8.00	0.639	1
ramA	9	4.57	21	3.82	0.673	1
adeN	1	0.51	1	0.18	0.458	0.840
adeI	1	0.51	3	0.55	1	1
adeJ	1	0.51	3	0.55	1	1
adeK	1	0.51	3	0.55	1	1

* *P*-values were corrected for multiple testing with the Benjamini-Hochberg method.

### 3.3. AMR genes and carbapenem resistance

**Figure 5:**
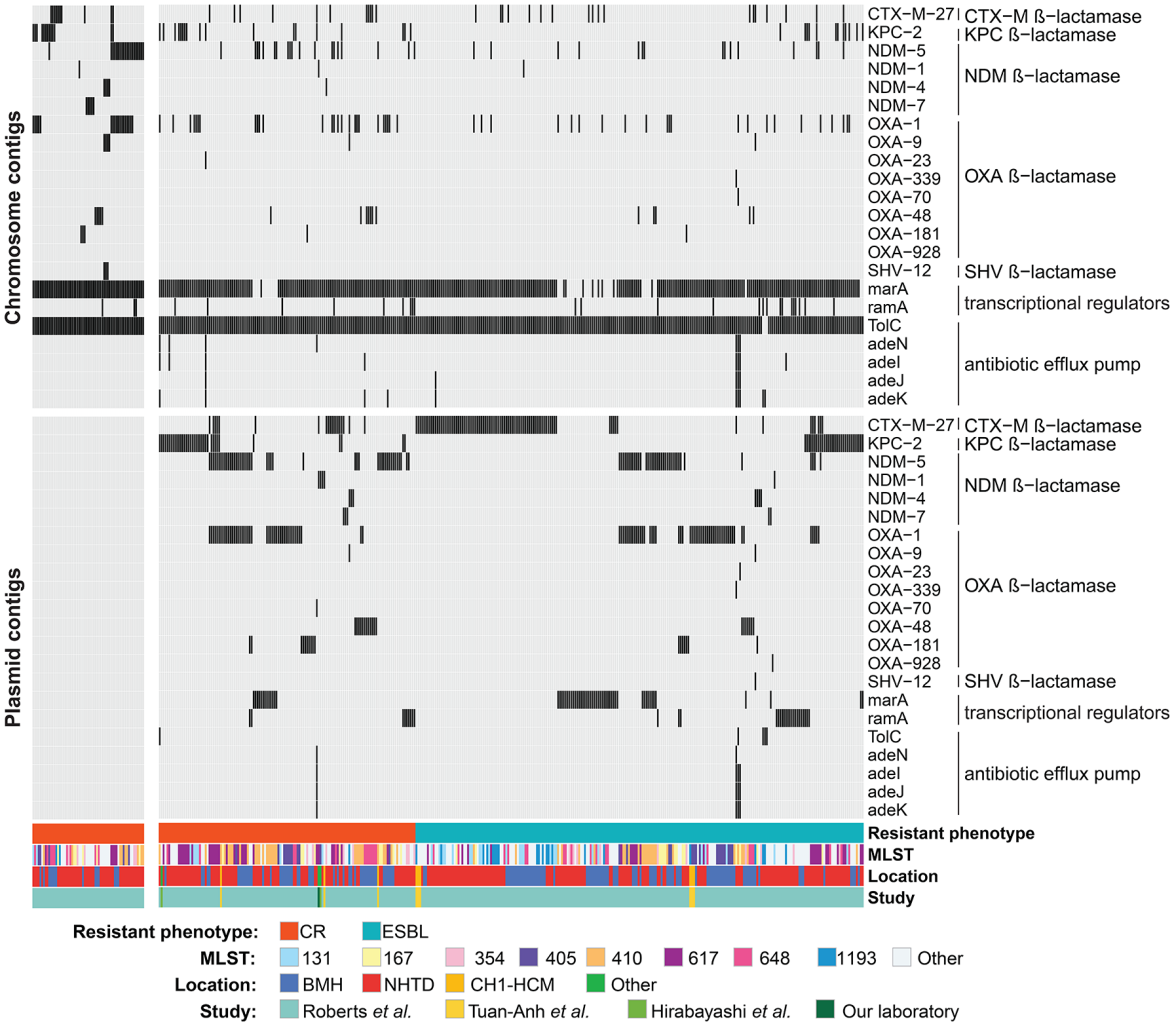
Carbapenem resistance (CR) genes on putative plasmid and chromosome contigs across isolates. All isolates carrying at least one carbapenem resistance gene on putative plasmid/chromosome contigs are shown on the right, whereas CR isolates without any carbapenem resistance genes on plasmid/chromosome contigs are on the left heatmap. Black and gray colors indicate presence and absence of AMR genes, respectively. The color bars at the bottom of the heatmaps indicate the resistance phenotype, sequence type (MLST), location and study from which the sequencing data of the isolates were taken. BMH, Bach Mai Hospital; NHTD, National Hospital for Tropical Diseases; CH1-HCM, Children’s Hospital 1-Ho Chi Minh city.

Then, the analysis focused on 22 AMR genes that are associated with resistance to carbapenems. AMR genes belonging to the efflux pump and β-lactamase families were found in both CR and ESBL-producing isolates. Efflux pump genes (*TolC, adeN, adeI, adeJ* and *adeK*) were found in <1% of the studied strains. *MarA* and its homolog *ramA*, which encode bacterial transcriptional activators involved in regulating the AcrAB/TolC multidrug efflux pump, and porin genes were detected in ~11% of isolates. Carbapenemase-encoding genes (*bla*_CTX-M_, *bla*_KPC_, *bla*_NDM_ and *bla*_OXA_) were detected in CR isolates and also in ESBL-producing isolates. Specifically, *bla*_CTX-M-27_, a β-lactamase-encoding gene that leads to resistance to carbapenems, cephalosporins, and penams, was the most frequently detected CR gene in putative plasmids (9.64% of CR and 15.82% of ESBL-producing isolates). Its presence in plasmids could be responsible for high resistance to antibiotics in CR and ESBL-producing isolates. Moreover, the frequency of *bla*_KPC-2_, *bla*_NDM-5_, *bla*_OXA-1_, *bla*_OXA-48_, and *bla*_OXA-181_ (β-lactamase-encoding genes) on putative plasmid contigs was higher in CR than ESBL-producing isolates (Fisher’s exact test: adjusted *p*-values <0.05). These genes are probably strongly associated with carbapenem resistance (Figure 5; Table 1). Additionally, >90% (*n* = 178/197) of the studied CR isolates carried at least one copy of the *bla*_KPC_, *bla*_NDM_, or *bla*_OXA_ gene in plasmid or chromosome contigs. Specifically, 67 of these CR isolates carried these genes only on plasmid contigs, 60 only on chromosome contigs, and 51 on both plasmid and chromosome contigs. Together, these findings indicated that carbapenem hydrolysis by KPC, NDM, and OXA carbapenemases is the major mechanism underlying carbapenem resistance in *E. coli* isolates with a significant contribution from plasmids.

### 3.4. Highly conserved structural organization of plasmid-encoded β-lactamase genes

**Figure 6:**
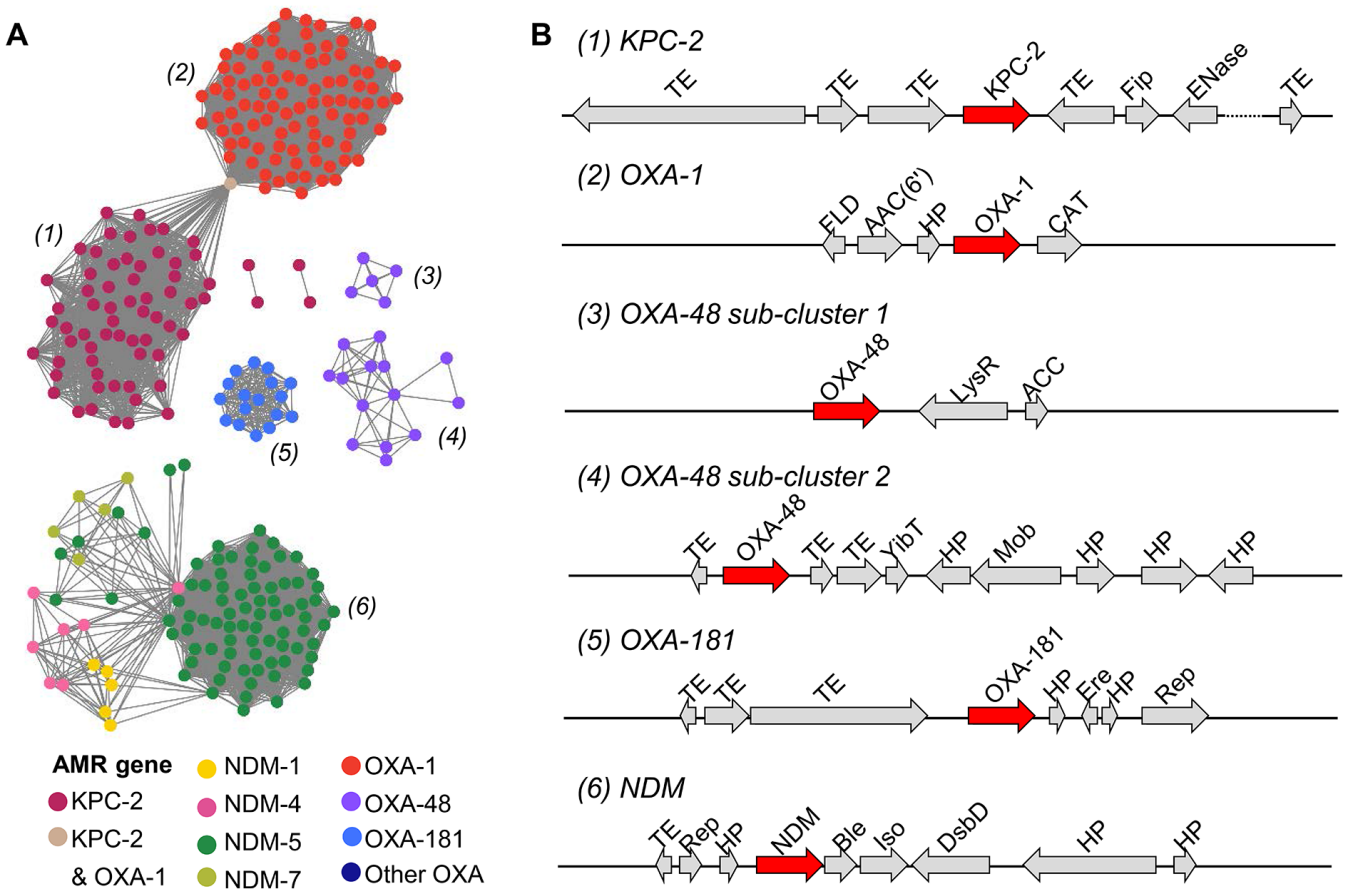
Characterization of putative plasmid contigs containing genes encoding the KPC, NDM and OXA β-lactamases in *E. coli* isolates. **(A)** Sequence similarity network of 304 plasmid contigs harboring *bla*_KPC_, *bla*_NDM_, and *bla*_OXA_ genes. Pairwise alignments between contigs were done with BLASTn, reciprocal BLAST, and an E-value threshold of 1 × 10^−10^. Each node represents a plasmid contig and is colored in function of the carried gene. Each plasmid contig cluster is labeled with a number. **(B)** The most frequent genetic organization of β-lactamase-encoding genes in plasmid contig clusters. Gene products from the RAST annotation are shown. TE, transposable element; Fip, filamentous phage production; ENase, plasmid conjugative transfer endonuclease; FLD, S-(hydroxymethyl) glutathione dehydrogenase; AAC(6′), aminoglycoside 6′ − N − acetyl transferase type Ib − cr; HP, hypothetical protein; CAT, chloramphenicol O-acetyltransferase; LysR, LysR transcriptional regulator; ACC, acetyl-coenzyme A carboxyl transferase, YibT, uncharacterized protein YibT; Mob, mobilization protein; Ere, erythromycin esterase; Rep, DNA replication protein; Ble, bleomycin resistance gene; PRAI, phosphoribosylanthranilate isomerase; DsbD, cytochrome C-type biogenesis protein DsbD.

Next, a sequence similarity network was built using 304 putative plasmid contigs that harbored *bla*_KPC_, *bla*_NDM_ and *bla*_OXA_ genes to obtain a global view of the genetic organization of β-lactamase-encoding genes on plasmids. Plasmid contigs carrying the same β-lactamase-encoding gene shared a high degree of genetic similarity (sequence identity >99% and minimum query coverage of 90%) in pairwise alignments. All contigs carrying different *bla*_NDM_ genes were grouped into one cluster. Contigs carrying different *bla*_OXA_ genes showed a great diversity of genetic structures and were organized in four clusters. Moreover, plasmid contigs harboring *bla*_OXA-48_ were classified in two sub-clusters. Although the clusters of the plasmid contigs carrying *bla*_OXA-1_ and *bla*_KPC-2_ did not share any sequence similarity, they were connected through a contig that contained both the *bla*_OXA-1_ and *bla*_KPC-2_ genes (Figure 6A). The genetic environment surrounding β-lactamase-encoding genes was similar in CR and in ESBL-producing isolates. In agreement with the result of the sequence similarity network, the structural organization of β-lactamase-encoding genes within each cluster was highly conserved across plasmid contigs (Figure 6B). Transposable elements (TEs; including transposons and insertion sequences), which play important roles in the spread of AMR genes, particularly *bla*_KPC_, *bla*_NDM_ and *bla*_OXA_, in healthcare settings (Partridge et al., 2018), were often located close to the β-lactamase-encoding genes.

## 4. Discussion

The emergence of ESBL-producing and CR *Enterobacteriaceae* is a public health threat worldwide, including Vietnam, because the latest generation of β-lactam antibiotics would become ineffective against infectious diseases caused by these species. Importantly, AMR *Enterobacteriaceae* are spreading not only in hospital settings, but also in the communities and in agricultural settings (Carrique-Mas et al., 2020). Dissemination of AMR genes by mobile genetic elements, especially plasmids, is considered the main mechanism of the emergence of drug resistance in bacteria (Partridge et al., 2018). In this study, the plasmid assemblies of 751 multidrug-resistant *E. coli* from hospitals in Vietnam were generated using short-read sequencing data from four studies in Vietnam. Overall, the studied isolates contained many putative plasmids that harbored a great diversity of AMR genes, particularly carbapenemase-encoding genes.

### 4.1. Genetic similarity of plasmids

The BLASTn homology search of contigs (assembled with plasmidSPAdes) against the NCBI RefSeq plasmid database and *E. coli* complete genomes (to determine the closest relative putative plasmid contigs) showed that apart from *E. coli* origin, putative plasmid contigs were found to be originated from other *Enterobacteriaceae* species or from the *Vibrionaceae* and *Moraxellaceae* families. More importantly, despite their different species/genus origin, theplasmid homologs shared high genomic similarity, which is broadly in agreement with a previous study on the prokaryotic plasmidome (Redondo-Salvo et al., 2020). These findings suggest that genetic exchange events by plasmid conjugation occur frequently within the *Enterobacteriaceae* family, especially in *E. coli* species but also between species. The potential plasmidic horizontal transfers between bacterial species, e.g., *A. baumanni* and *E. coli* have been reported (Chatterjee et al., 2017; Cooper et al., 2017). This aspect is fundamental to explain the ecology of AMR and to understand the emergence of drug resistance in the different bacterial species of public health interest.

### 4.2. Molecular epidemiology of CTX-M β-lactamases

ESBL-producing and CR isolates harbored many different AMR genes on their plasmids. The most frequently detected genes encoded antibiotic efflux pumps that eliminate antimicrobial agents from host cells (Kumar et al., 2020). Genes encoding CTX-M β-lactamases also were frequently identified on plasmids of ESBL-producing and CR isolates, as already previously observed in clinical ESBL-producing *E. coli* isolates (Ma et al., 2005; Ghenea et al., 2022). Strikingly, each CTX-M variant is widespread over a particular geographic area, thus providing epidemiological evidence for the transmission of β-lactamase-encoding genes within and between communities (Canton and Coque, 2006; Rossolini et al., 2008). In the present study, the *bla*_CTX-M-27_, *bla*_CTX-M-15_ and *bla*_CTX-M-55_ genes were widely represented on plasmids of ESBL-producing and CR strains. Previous studies showed that the genes encoding these CTX-M variants are predominant in ESBL-producing *E. coli* isolates from humans and farm animals in Vietnam (Biedenbach et al., 2014; Nguyen et al., 2019). CTX-M-15 is the dominant CTX-M variant worldwide. Other CTX-M variants (e.g., CTX-M-55, CTX-M-27) that were initially specific to some world regions have now spread globally due to international travel (Bevan et al., 2017; Nakayama et al., 2020). Thus, monitoring the emergence of plasmid-carried *bla*_CTX-M_ genes, particularly genes encoding new variants, could provide important information for developing strategies to reduce the transmission of AMR genes via plasmids in clinical settings.

### 4.3. Molecular epidemiology of carbapenemases

Plasmid-carried carbapenemase-encoding genes are considered the main cause of the resistance phenotype in CR *E. coli*. In our analysis, genes encoding the KPC-2, NDM-5, OXA-1, OXA-48, and OXA-181 β-lactamases were more frequently detected on plasmids in CR than ESBL-producing isolates. The *bla*_KPC_ gene was first detected on a plasmid of a carbapenem-resistant *K. pneumoniae* strain in the southern United States in 2001 (Yigit et al., 2001). It has then rapidly spread to other bacterial pathogens, including *E. coli,* worldwide, including in Vietnam (Petrella et al., 2008; Woodford et al., 2008; Richter et al., 2011; Linh et al., 2021). Similarly, plasmid-carried *bla*_NDM-5_ was identified in patients in the United Kingdom and the United States (patient hospitalized in India) in 2016–2017 (Hornsey et al., 2011; Rojas et al., 2017), and in multidrug-resistant clinical *E. coli* strains in many countries (Pitart et al., 2015; Soliman et al., 2016; Zou et al., 2020). In Vietnam, plasmid-encoded *bla*_NDM-1_ was detected in clinical *Enterobacteriaceae* isolates from a surgical hospital in 2010 (Tran et al., 2015). To our knowledge, Roberts et al. (2022) were the first to describe the presence of *bla*_NDM-5_ in clinical isolates in Vietnam. In our study, plasmid-carried *bla*_NDM-1_, but not *bla*_NDM-5_, was detected in the XP817 and MH-13 isolates collected in 2012–2013, whereas plasmid-carried *bla*_NDM-5_ was detected in clinical samples isolated in 2017–2019. This suggests the emergence of plasmid-carried *bla*_NDM-5_ in Vietnam approximately in 2017, close to the year of its first report in other countries.

Plasmid-encoded OXA β-lactamases can hydrolyze β-lactam substrates, including carbapenems (Evans and Amyes, 2014). The KPC, NDM and OXA enzymes can mediate carbapenem resistance. Their hydrolytic activity depends on the expression level of the genes encoding these enzymes that is mostly influenced by their genetic environment and copy number (Roth et al., 2011; Evans and Amyes, 2014; Paul et al., 2017). This could explain why some ESBL-producing isolates that harbor these β-lactamase-encoding genes do not show any phenotypic resistance to carbapenems.

The sequence similarity network based on pairwise alignments of plasmid contigs harboring the *bla*_KPC_, *bla*_NDM_ and *bla*_OXA_ genes captured a high degree of similarity between contigs carrying the same AMR gene. This confirmed the results by Roberts et al. (2022) showing that AMR genes are transmitted within and between hospital settings in Vietnam. The genetic environment around the *bla*_KPC-2_ and *bla*_NDM_ genes is largely consistent with what reported by previous studies (Dortet et al., 2012; Cuzon et al., 2013; Martins et al., 2020). Specifically, our analysis showed that *bla*_KPC-2_ is located between two TEs, whereas *bla*_NDM_ genes are close to the bleomycin resistance gene located downstream of a TE and share the same promoter. Conversely, *bla*_OXA_ genes were found in several clusters with different environmental structures, as previously reported by Evan & Amyes (Evans and Amyes, 2014). This could be explained by spontaneous DNA rearrangements following acquisition of the *bla*_OXA_ by horizontal transfer.

### 4.4. Limitations

Our study, based on short-read whole-genome sequencing data, provided general and predictive information on plasmid profiles in 751 ESBL-producing and CR *E. coli* isolates. This work has certain limitations. We could only assemble partially plasmids from short-read data using the existing assembly tool and therefore part of plasmid information remains unknown. Another limitation of our study was sample isolation bias since most of the studied strains were isolated from patients of two hospitals in northern Vietnam. Nevertheless, the current study serves as a good foundation for further analysis on a larger scale to have a more detailed look into resistance plasmid epidemiology at the national and global level.

## 5. Conclusion

This study highlights the high potential of horizontal transfer and dissemination of AMR genes encoded by plasmids in multidrug-resistant *E. coli* isolates in Vietnam. These putative plasmids could be transferred among different *Enterobacteriaceae* species and genera. The number of plasmid-carried AMR genes was higher in CR than in ESBL-producing isolates, especially β-lactamase-encoding genes. The genetic environment surrounding carbapenemase-encoding genes in plasmid contigs was highly conserved with the presence of transposable elements that might facilitate their spread in microbial communities.

## Data availability statement

The datasets presented in this study can be found in online repositories. The names of the repository/repositories and accession number(s) can be found in the article/Supplementary material.

## Ethics statement

Ethical review and approval was not required for the study on human participants in accordance with the local legislation and institutional requirements. Written informed consent for participation was not required for this study in accordance with the national legislation and the institutional requirements.

## Author contributions

QHN and TTTT: study concept and design. TTHL, STN, K-OTN, and DVQ: data acquisition. QHN, TTHL, STN, and TTTT: data analysis and interpretation. QHN, TTHL, STN, JH, A-LB, and TTTT: drafting of the manuscript. All authors contributed to the article and approved the submitted version.

## Funding

This work was financially supported by funding from the USTH institutional S&T project (USTH.LS.01/21-22). QHN was funded by the Postdoctoral Scholarship Programme of Vingroup Innovation Foundation (VINIF), code VINIF.2022.STS.58.

## Conflict of interest

The authors declare that the research was conducted in the absence of any commercial or financial relationships that could be construed as a potential conflict of interest.

## Publisher’s note

All claims expressed in this article are solely those of the authors and do not necessarily represent those of their affiliated organizations, or those of the publisher, the editors and the reviewers. Any product that may be evaluated in this article, or claim that may be made by its manufacturer, is not guaranteed or endorsed by the publisher.

## Abbreviations

**Abbreviations**
AMR: antimicrobial resistance
CR: carbapenem-resistant
MDR: multidrug-resistant
ESBL: extended spectrum beta-lactamase
TE: transposable element
