# Supplementary material for: Large-scale analysis of putative plasmids in clinical multidrug-resistant Escherichia coli isolates from Vietnamese patients
Source: Front Microbiol. 2023 May 31;14:1094119. doi: 10.3389/fmicb.2023.1094119 (PMC10265513; doi:10.3389/fmicb.2023.1094119)
Supplement: Supplementary file 1 [file Image_1.PDF]

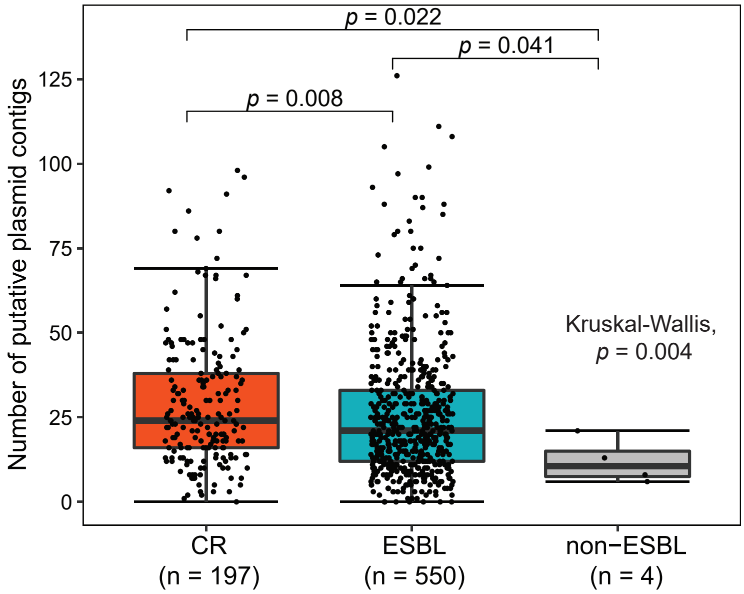

Supplementary Figure S1. Boxplot shows the distributions of contigs from the plasmidSPAdes assembler in carbapenem-resistant (CR) isolates, extended-spectrum beta-lactamase (ESBL)-producing isolates, and non-ESBL-producing isolates; n, number of isolates. Distributions were compared with the Kruskal-Wallis test followed by the Dunn's post-hoc test and corrected for multiple testing with the Benjamini-Hochberg method.
