# Supplementary material for: Large-scale analysis of putative plasmids in clinical multidrug-resistant Escherichia coli isolates from Vietnamese patients
Source: Front Microbiol. 2023 May 31;14:1094119. doi: 10.3389/fmicb.2023.1094119 (PMC10265513; doi:10.3389/fmicb.2023.1094119)
Supplement: Supplementary file 2 [file Image_2.PDF]

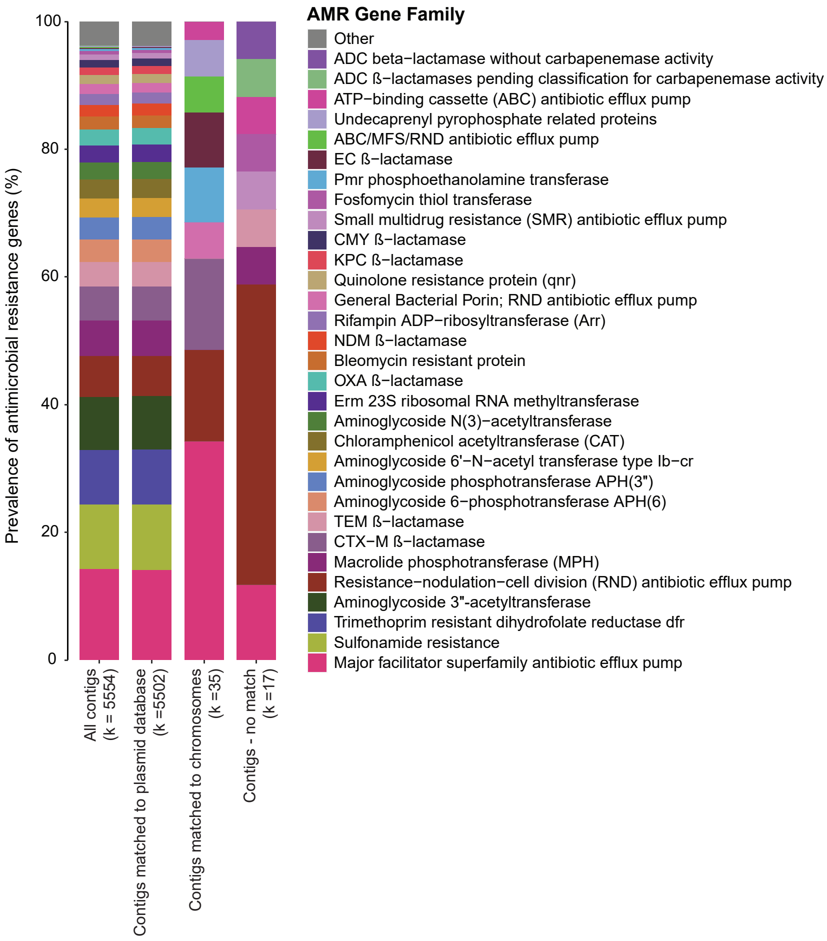

Supplementary Figure S2. Frequency of antimicrobial resistance (AMR) gene classified into AMR gene families in all contigs assembled with plasmidSPAdes assembler, contigs matched to the plasmid database, contigs matched to chromosomes, and contigs without homologs (no match). AMR genes with frequency <1% were merged into the "Other" group. k, total number of AMR genes across contigs.
